# Representation of probabilistic outcomes during risky decision-making

**DOI:** 10.1038/s41467-020-16202-y

**Published:** 2020-05-15

**Authors:** Giuseppe Castegnetti, Athina Tzovara, Saurabh Khemka, Filip Melinščak, Gareth R. Barnes, Raymond J. Dolan, Dominik R. Bach

**Affiliations:** 1 Computational Psychiatry Research, Department of Psychiatry, Psychotherapy, and Psychosomatics, University of Zurich, Zurich, Switzerland; 2 Neuroscience Centre Zurich, University of Zurich, Zurich, Switzerland; 3 Institute of Cognitive Neuroscience, University College London, London, UK; 4 Department of Computer Science & Faculty of Medicine, University of Bern, Bern, Switzerland; 5 Helen Wills Neuroscience Institute, University of California, Berkeley, USA; 6 Wellcome Centre for Human Neuroimaging, University College London, London, UK; 7 Max Planck UCL Centre for Computational Psychiatry and Ageing, University College London, London, UK

**Keywords:** Decision, Reward

## Abstract

Goal-directed behaviour requires prospectively retrieving and evaluating multiple possible action outcomes. While a plethora of studies suggested sequential retrieval for deterministic choice outcomes, it remains unclear whether this is also the case when integrating multiple probabilistic outcomes of the same action. We address this question by capitalising on magnetoencephalography (MEG) in humans who made choices in a risky foraging task. We train classifiers to distinguish MEG field patterns during presentation of two probabilistic outcomes (reward, loss), and then apply these to decode such patterns during deliberation. First, decoded outcome representations have a temporal structure, suggesting alternating retrieval of the outcomes. Moreover, the probability that one or the other outcome is being represented depends on loss magnitude, but not on loss probability, and it predicts the chosen action. In summary, we demonstrate decodable outcome representations during probabilistic decision-making, which are sequentially structured, depend on task features, and predict subsequent action.

## Abstract (web summary)

Goal directed behavior requires the sequential retrieval and evaluation of the multiple choices for action and their deterministic outcomes. Here, the authors report sequential, decodable probabilistic outcome representations in magnetoencephalography (MEG) signals during a risky foraging task.

## Introduction

Thinking before acting is a prerequisite of wise choices, most educators would say. The cognitive instantiation of this notion in goal-directed behaviour is the prospective evaluation and subsequent comparison of the available options, before selecting appropriate actions^1–5^. To elucidate the neural underpinnings of prospective outcome evaluation, extensive research has investigated which brain networks represent the possible outcomes of an action, establishing contributions from various prefrontal areas and a prominent role for the orbitofrontal cortex (OFC)^6–12^. In many naturalistic environments, action-outcome transitions are probabilistic. In this case, goal-directed choices require retrieval of multiple possible action outcomes to compute expected action values^4,13–15^. How this is instantiated in neural circuits remains elusive. Here, we test the hypothesis that these multiple outcomes are retrieved sequentially.

Our hypothesis is based on a similarly structured and well-studied problem: the choice between multiple actions with deterministic outcomes. In this case, multiple outcomes need to be evaluated as well, although they must be compared rather than integrated. In this case, there is a body of evidence for sequential outcome retrieval. First, manipulating differential attention to the outcomes during deliberation affects choice^16–18^. This led to the hypothesis that (internal or external) attentional focus biases choice towards the attended option^19,20^, which is supported by reports of selective representation of the attended value^21–24^. Independent of this attentional mechanism, animal electrophysiology^10–12,25^ and human neuroimaging^6,15,26–29^ suggest that neural outcome representations are reinstated during choice deliberation, and this process has been proposed to be sequential^30^. Further support to the sequential structure of outcome evaluation comes from research on spatial navigation. At spatial choice points, rodent hippocampi reinstate the different trajectories to remembered goals one after the other; which trajectory is reinstated more often predicts immediate future behaviour^31,32^.

In summary, a large body of literature suggests that choice between multiple deterministic actions is at least partly based on sequential outcome retrieval across species, even though the neuronal mechanism of the ensuing choice is under debate^33^. From a computational point of view, sequential representation may be advantageous because it scales to larger number of options and avoids computational inaccuracy deriving from attempting to represent all options simultaneously^3^.

Here, we hypothesised that the same would also be the case for evaluation of probabilistic outcomes following a single action in a biologically relevant scenario. We used a previously established loss/reward decision-making task embedded in a grid-world approach/avoidance conflict computer game^34–36^. The task mimics the natural scenario of foraging under predation risk and may, therefore, be particularly relevant to understand biological decision-making^37–39^. Since evaluation of multiple action outcomes is required only in model-based decisions, and some avoidance actions appear to be habitual^40^, we note that there is evidence for at least partly model-based control in foraging under predation^38,39^, including the particular task we use here^34,35^. In this task, a human agent can decide whether, and how rapidly, to approach a spatial location to obtain a constant reward, under risk of being virtually attacked by a predator and incurring a variable loss^34,36^. Loss probability and magnitude are manipulated independently. With this task, we sought to assess the existence of sequential outcome representations during choice deliberation. Given the effect of internal attention on deterministic choice^3,19^, we further hypothesised that which outcome was being represented more often could be influenced by task features, and relate to the ensuing action.

To address these questions, we harnessed the temporal resolution of magnetoencephalography (MEG), which has been successfully used to decode off-line replay of action trajectories in humans^41,42^. We followed this approach to decode outcome representations by multivariate analysis of MEG sensor signals. Although much previous work on deterministic action outcomes and their values has focused on orbitofrontal cortex^6,10–12,27^, there is evidence for widespread cortical representation of anticipated and experienced outcome values^13,43–45^, such that we did not spatially constrain our analysis.

## Results

**Fig. 1:**
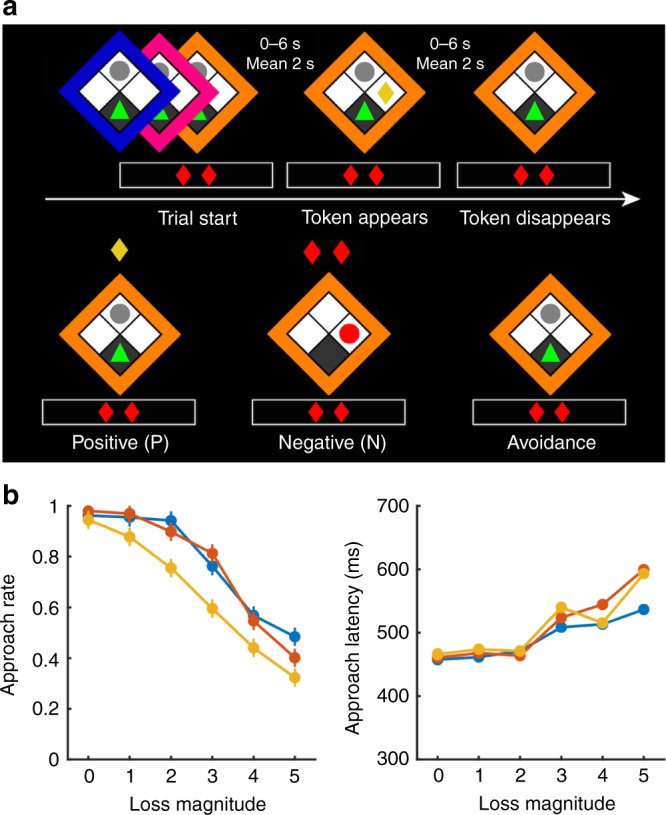
Approach/avoidance conflict task. **a** Top: Loss probability (indicated by the frame colour) and magnitude (number of red diamonds) are shown at trial start, while reward magnitude is always one token. The player (green triangle) is located at the safe position at the bottom corner of the grid and an inactive predator (grey circle) is located at the top corner. After a random time interval, a token appears either on the left or the right side, and disappears after another random time interval. Bottom: If the player leaves the safe position to approach the token, one of three outcomes ensues: a positive outcome P, if the player collects the token and safely returns to the initial position; a negative outcome N, if the predator wakes up and catches the player, causing the loss of a number of tokens; or a rare neutral outcome (not shown), in which the token disappears before the player can reach it. Alternatively, the player can decide to not perform an action and avoid the token. The trial ends 1000 ms after the disappearance of the token. **b** Behavioural results. Approach rate (left) and average approach latency (right) for each loss probability and magnitude; error bars represent the standard error of the mean (s.e.m.). See Table 1 for inference statistics.

We recorded MEG while participants played an approach/avoidance conflict computer game^36^ (Fig. 1a). On each trial, a reward token appeared. Collecting the token (approach choice) entailed a small probability that the player was caught by a virtual predator (loss probability: low, medium, high). This probability was signalled by the frame colour and learned by experience beforehand (Fig. 1a). Being caught caused the loss of a variable number of reward tokens, which was explicitly signalled at the bottom of the screen (loss magnitude: 0–5 tokens). Loss probability and magnitude were randomly balanced on a trial-by-trial basis. At the end of the game, cumulative earnings from six randomly selected trials were paid out at a rate of 6 GBP per collected token. Behavioural results are summarised in Table 1 (ref. ^36^). Participants were more likely to approach when loss probability and loss magnitude were smaller (Fig. 1b). Smaller loss probability and magnitude also resulted in shorter approach latency (Fig. 1b), although, notably, this is not reward-maximising under task instructions^34,35^.

**Table 1:** Effect of loss manipulations on behaviour (approach action, and approach latency).

	*F*	*p*	df
Approach action			
Loss probability	27.85	<0.001	2; 12,380
Loss magnitude	438.78	<0.001	5; 12,380
Loss probability x loss magnitude	7.18	<0.001	10; 12,380
Approach latency			
Loss probability	3.54	0.029	2; 9,082
Loss magnitude	20.84	<0.001	5; 9,082
Loss probability x loss magnitude	2.29	0.019	10; 9,082

We show fixed-effects *F*-tests from a loss probability x loss magnitude (generalised) linear mixed effects model with random subject intercept.

**Fig. 2:**
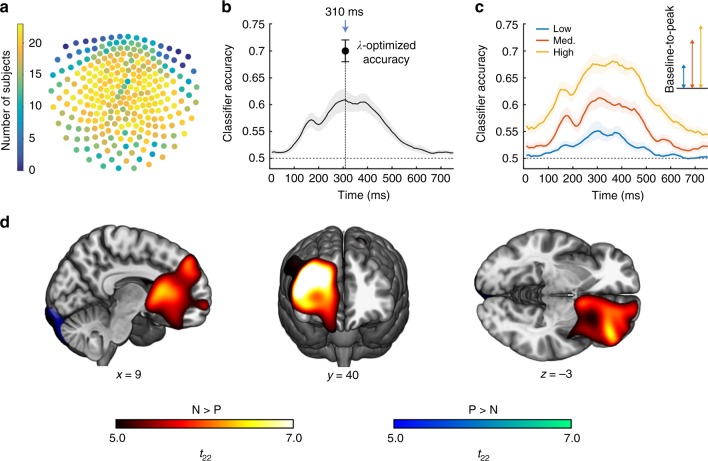
Classifier construction. **a** Distribution of the participant-specific set of 135 sensors that contained the fewest eye blinks. Warm-coloured sensors were retained more frequently. **b** Average balanced classification accuracy (thick black line) with its standard error of the mean (grey shade), obtained from *n* = 21 participants, as a function of time elapsed after outcome onset (*t* = 0). For selection of the post-outcome time bin, the Lasso coefficient *λ* was set arbitrarily to 0.025. At the resulting peak time bin (310 ms), *λ* was then optimised to build the classifier used for decoding, resulting in an average balanced accuracy of 0.70 ± 0.02 (mean ± s.e.m.; black dot). **c** Same as in **b**, but after dividing the training set according to loss probability. Classification accuracy is particularly low when negative outcomes are rare (low). Inset: comparison of the baseline-to-peak accuracy at 310 ms for each threat probability. **d** Source reconstruction of brain activity around the time bin used for building the classifier (310 ms), computed with a beamforming algorithm on an interval of 100 ms duration. Figure shows brain regions where broadband oscillatory power (1–50 Hz) was higher for the negative outcome (N, warm colours) or for the positive outcome (P, cold colours). Results are corrected for whole-brain family-wise error (FWE) at *p* < 0.05. Copyright (C) 1993–2004 Louis Collins, McConnell Brain Imaging Centre, Montreal Neurological Institute, McGill University.

Next, we sought evidence for outcome representations. To this end, we trained participant-specific multivariate classifiers to distinguish the field patterns elicited at the MEG sensors after participants encountered the outcomes. To minimise an impact of eye blinks, we followed a previous approach^41^ and selected the participant-specific set of 135 MEG channels that contained the smallest amount of artefacts (Fig. 2a). Retaining all channels followed by topography-based artefact-correction in a supporting analysis (which is similar to ICA-based artefact-correction)^46^ yielded very similar results. Classifiers were trained to distinguish the two frequent outcomes that could follow an approach action: N (negative: participant caught) or P (positive: token collected). Neutral outcomes of this action (token missed) were overall rare (Table 2) and not analysed. We first trained classifiers separately for each 10-ms time bin during a 0–750 ms interval after onset of outcome presentation and examined the temporal profile of classification performance in terms of balanced accuracy. We chose balanced accuracy as a metric because the number of P and N exemplars was unequal. Regardless of the relative number of samples in the training set, chance level for balanced accuracy is 0.5, as this score is computed as the average proportion of correct classification for each of the two outcomes. This provides more reliable accuracy estimates for classifiers built on unbalanced datasets^47^. Group-level balanced accuracy peaked around 300 ms after outcome onset (Fig. 2b), implying that MEG field patterns at this time point provided maximal discrimination between P and N. We then optimised the participant-specific regularisation coefficient *λ* of the logistic regression (Supplemental Fig. 1) to build the final pattern classifiers based on data from this time bin (see Fig. 2a for the distribution of channels contributing to this classification). For these classifiers, cross-validated peak accuracy was 0.70 ± 0.02 (mean ± s.e.m.). Since the negative outcome N was much rarer than the positive outcome P, and electromagnetic brain activity 310 ms after an event is observed after oddballs (i.e., rare events^48^) we sought to explore whether classification was indeed capturing a neural response to outcome identity, or instead a surprise signal associated with the rarer event. To this end, we reasoned that if our classifier was capturing a surprise signal, this would result in higher classification accuracy when the negative outcome is rarer (i.e., more surprising), as was the case with lower loss probabilities. We thus divided the training set according to loss probability and trained separate classifiers for each set. We found that baseline-to-peak classification accuracy was higher in the context of higher loss probability (Fig. 2c). This pattern is not consistent with a surprise-related explanation of the classification and supports the notion that we are classifying outcomes based on their identity.

**Table 2:** Outcome distribution.

0	1	2	3	4	5
Low					
P: 24.3 ± 2.4	P: 25.0 ± 3.1	P: 24.7 ± 2.9	P: 19.7 ± 8.6	P: 14.7 ± 10.4	P: 12.7 ± 10.7
N: 2.9 ± 1.4	N: 2.3 ± 1.1	N: 1.9 ± 1.6	N: 1.5 ± 1.2	N: 1.2 ± 1.2	N: 0.7 ± 1.1
F: 1.6 ± 1.3	F: 1.3 ± 1.2	F: 1.7 ± 1.2	F: 1.7 ± 1.4	F: 1.1 ± 1.1	F: 1.1 ± 1.5
Medium					
P: 23.0 ± 2.3	P: 23.0 ± 3.1	P: 21.8 ± 4.3	P: 19.5 ± 6.5	P: 13.1 ± 9.4	P: 9.9 ± 9.2
N: 4.4 ± 2.1	N: 4.3 ± 2.2	N: 4.1 ± 2.3	N: 3.7 ± 2.1	N: 2.2 ± 2.2	N: 1.4 ± 2.1
F: 2.0 ± 1.4	F: 1.7 ± 1.6	F: 1.1 ± 1.0	F: 1.2 ± 0.9	F: 1.1 ± 1.0	F: 0.7 ± 1.1
High					
P: 18.9 ± 2.7	P: 17.9 ± 5.2	P: 16.6 ± 7.6	P: 12.9 ± 8.4	P: 9.7 ± 8.7	P: 7.0 ± 7.7
N: 8.1 ± 2.7	N: 7.2 ± 3.3	N: 5.0 ± 2.9	N: 3.7 ± 3.1	N: 3.1 ± 2.8	N: 2.0 ± 2.6
F: 1.4 ± 1.2	F: 1.3 ± 1.0	F: 1.1 ± 0.9	F: 1.3 ± 1.4	F: 0.5 ± 0.7	F: 0.6 ± 0.9

Listed are the occurrences of each outcome for approach trials (mean ± standard deviation; P: positive; N: negative; F: failed) for each combination of loss probability (low, medium, high) and magnitude (0–5).

To build the classifier, we had collapsed across all loss probability and magnitude conditions. Previous work has shown that salient manipulations of the context, as well as the associative structure of outcome predictions, can affect the coding scheme under which outcomes are represented^10,11,49^. Thus, it is possible that successful classification is specific within experimental conditions and does not generalise. To explore this possibility, we employed a cross-classification approach: we trained data on all loss probability or magnitude levels except one and tested the classifier on the left-out condition. In this way, the classifiers had never seen the loss probability or magnitude level they were being tested on. Even in this case, the classifiers’ performance remained robustly above chance (Supplemental Fig. 2) and on the same order of magnitude as the performance obtained with all conditions collapsed in the same training set (Fig. 2b). This suggests that our classifier is based on features of the outcome representation that are shared between different experimental conditions.

To explore the neural regions that most likely generated the MEG field patterns that contribute to outcome classification, we used a beamforming approach to reconstruct the most likely neural sources of the sensor-level MEG data. Source activity was reconstructed within a temporal window centred at the peak of the classification performance (i.e., 310 ms post-outcome), and with 100 ms duration. As a result, we found stronger source-level activity during presentation of (rarer) negative compared to positive outcomes predominantly in a large cluster centred the right dorsolateral PFC and extending to the OFC (peak voxel in MNI space: [30,38,40], *t*_22_ = 8.22, *p* < 0.001 whole-brain corrected for FWE; Fig. 2d) while a smaller source centred between the visual cortex and the cerebellum displayed the opposite pattern (i.e., *P* > *N;* [−8, −98, −22], *t*_22_ = 6.39; *p* < 0.010). It therefore appears likely that the classifiers predominantly captured differential activity in prefrontal regions, including OFC and dorsolateral prefrontal cortex (dlPFC), which have been often implicated in the representation of behavioural outcomes^6,11,12,27^, as well as visual areas^44,45^.

We then used these classifiers to decode MEG field patterns recorded during choice deliberation (Fig. 1a). Since the reward token appeared at a random time point during deliberation, we separately extracted data before and after token appearance. The first epoch (trial start) spanned 0–1500 ms after trial onset. Epochs during which the token appeared were discarded; this exclusion was independent from the experimental conditions by design. From a total of 540 epochs per participant, an average of 305 epochs were retained. Secondly, we analysed an epoch of 0–300 ms after token onset (token appearance) and discarded all epochs during which a movement occurred or the token had disappeared. An average of 522 epochs per participant were retained for this second analysis. Since approach latency depended on experimental condition (Fig. 1b), so did the exclusion of trials. However more than 93.3% of trials were retained for any individual experimental condition (Supplemental Fig. 3).

**Fig. 3:**
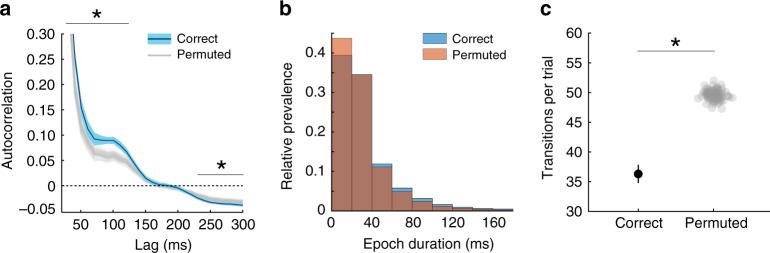
Temporal structure of the reconstructed representations. **a** Autocorrelation of the reconstructed probabilities (dark blue line) and its s.e.m. (light blue shade) obtained from *n* = 21 participants, against the mean autocorrelation obtained by repeating the entire analysis (*n* = 21) 100 times after randomly permuting the class labels (grey transparent lines). Stars indicate the time lags at which the autocorrelation differed from chance (two-sided cluster-level permutation test, *p* < 0.010). **b** Distribution of the duration of steady outcome representation epochs (blue) against the null distribution derived from permuted classifiers (red; two-sample Kolmogorov–Smirnov test, *p* < 0.001). **c** Consistently with the epoch durations in **b**, number of transitions between most likely outcome over the 1500 ms deliberation interval is lower than chance (permutation test, *p* < 0.010). The error bar represents the s.e.m. of the number of transitions for the correct classifier.

Our classifier assigned a probability of P or N representation to every time point. To verify that these decoded time series contained a neural representation of the action outcomes, we first tested whether their temporal structure deviated from chance. To do so, we computed the autocorrelation at different lags and compared it with the autocorrelation of time series decoded using classifiers built on permuted trial labels. This analysis was restricted to trial start epochs (0–1500 ms from trial start), whose longer duration allowed a better evaluation of the autocorrelation. We used cluster-level correction^50^ to test whether autocorrelation deviates from chance anywhere within the tested interval; the location of the effect is reported for illustration. We found that representation probability was more autocorrelated than chance for time lags up to ~150 ms (two-sided cluster-level permutation test, *p* < 0.010), and less than chance after 200 ms (*p* < 0.010; Fig. 3a). Next, we mapped the reconstructed probability at each deliberation time point into the outcome most likely to be represented (i.e., positive if *p*(P) > *p*_chance_; Negative if *p*(N) = 1 – *p*(P) > 1 – *p*_chance_), and analysed the duration of epochs of steady representation. The distribution of this duration was biased towards longer-lasting epochs, compared to chance (two-sample Kolmogorov–Smirnov test, *p* < 0.001; Fig. 3b). Consistent with this, the average number of representational transitions per trial was lower than chance (all 100 permuted classifiers produced a higher average number of transitions; i.e., *p* < 0.010; Fig. 3c).

Overall, these results indicate that during deliberation the outcome representations occur in epochs with longer-than-chance duration and tend to anticorrelate with outcome representations more than 200 ms apart. This suggests an alternating and thus sequential neural representation of the two possible action outcomes.

**Fig. 4:**
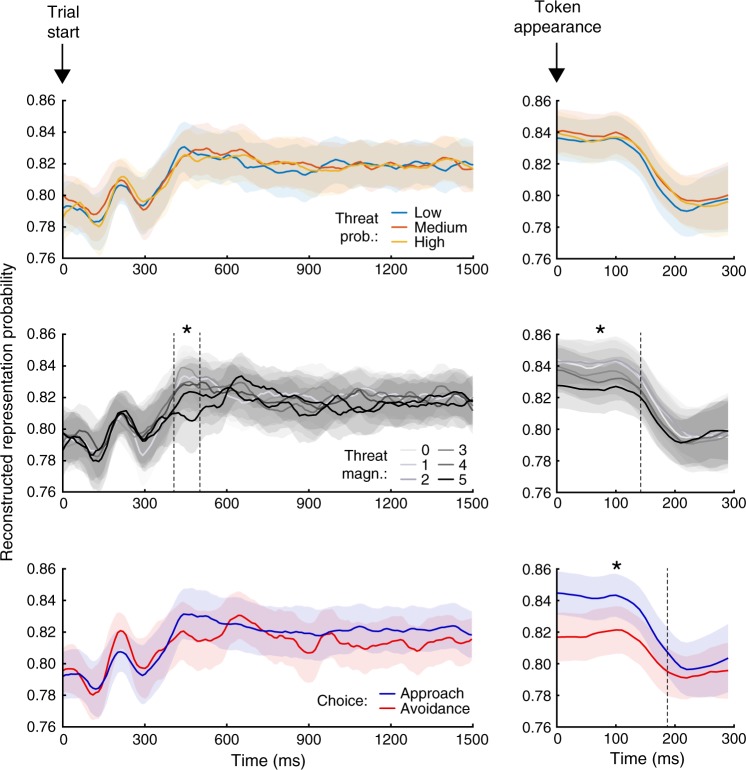
Decoded outcome representation probabilities. Displayed are the probabilities that the MEG field patterns during deliberation represents the positive outcome (*p*(Positive); *p*(Negative) = 1 – *p*(Positive)), for epochs between 0 and 1500 ms from trial start and between 0 and 300 ms from token appearance (note the different time scales in plots aligned to trial start or token appearance). The shaded areas indicate the s.e.m. of the corresponding probability; Intervals denoted with asterisk (*) illustrate epochs in which the effect of the corresponding manipulation was statistically significant in a cluster-level random permutation test across the entire epoch.

Next, we investigated whether outcome representations during deliberation depended on loss probability and loss magnitude, and whether they were predictive of subsequent choice. Our classifier returned for each time point a probability that the positive or negative outcome was represented. We evaluated whether the probability that either outcome was represented in the MEG activity patterns varied under different levels of loss probability, loss magnitude, or preceding approach or avoidance choice. To this end, we fitted a 3 × 6 × 2 (loss probability: low, medium, high; loss magnitude: 0–5; choice: approach, avoidance) linear mixed model to the decoded outcome representations at each time bin. We tested for statistical significance with a non-parametric permutation test at the cluster level. This test allows inferring whether an effect exists anywhere within the tested interval; the location of the effect is reported for illustration. Results are displayed in Fig. 4. Reflecting the unbalanced training set, decoded outcome probabilities are above 0.5 in favour of P throughout the analysed intervals (Table 2). At trial start, the probability of a positive rather than negative outcome representation was further increased with lower loss magnitude. This was evident between about 400 and 500 ms after trial start and up to 140 ms after token appearance. In contrast, there was no impact of loss probability, nor any interaction between probability and magnitude. Therefore, lower loss magnitude may bias towards representing the positive outcome.

Outcome representation was also related to upcoming choice. The probability of representing the positive (rather than negative) outcome was higher when participants chose to approach, rather than to avoid, around 0–200 ms after token appearance (Fig. 4). The effect was evident immediately after token onset, suggesting that participants had already committed to a choice at that time.

So far, we used a classifier trained to discriminate P vs. N. This constrained any MEG field pattern to be assigned to either of the two outcomes, even if neither were represented. Hence, the analysis above provided insights on the ratio of outcome representations but was agnostic about which of the two outcome representations was responsible for a change in this ratio. To investigate this, we created two additional classifiers for each participant, trained to distinguish either *P* or *N* from baseline. Baseline exemplars were randomly selected from the inter-trial interval at time bins during the 1000 ms preceding trial start.

Similarly to the above analysis, we sought to evaluate how the representation of each outcome was influenced by loss probability and magnitude, and by upcoming choice. During deliberation, the previously demonstrated effect of loss magnitude emanated preferentially from stronger representation of positive outcome (Supplemental Fig. 4). In particular, P vs. baseline, but not N vs. baseline, depended on loss magnitude in the same direction (i.e., more pronounced representation with lower loss magnitude) and in the same time interval (i.e., 400–500 ms) as what we observed in the P vs. N classification (Fig. 4). In contrast, after token appearance, representation of negative outcome was stronger when loss magnitude was higher or when participants chose to avoid (Supplemental Fig. 4). At the same time, this analysis indicated that approach was preceded by increased positive outcome representation about 1400 ms into the action selection interval, whereas no effect of experimental condition on N vs. baseline was found after token appearance.

## Discussion

In this study, we investigated how probabilistic action outcomes are represented during choice deliberation. Using human MEG recordings, we trained multivariate classifiers to discriminate patterns of neural activity that distinguished action outcomes when they occurred. The crucial features of these activity patterns were most likely generated in prefrontal and visual areas. We then used these classifiers to decode outcome representations while participants made choices, and found that they were structured in time, consistent with an alternating retrieval. Stable outcome representations appeared to be shorter than 150 ms, and longer than 40 ms. The finding of potentially alternating, and thus sequential, outcome retrieval resonates with sequential retrieval of multiple outcomes in deterministic choice. Furthermore, we found that decoded outcome representations are influenced by loss magnitude early during deliberation, whereas they predict the ensuing choice during late deliberation and immediately before the action.

During choice deliberation, neural outcome representations were more autocorrelated than chance at short time lags (i.e., <150 ms) and less than chance at longer time lags (i.e., >200 ms). Analysis of stable representation epochs revealed that epochs of >40 ms duration occurred more often than expected by chance alone. This suggests that outcome representations occur with characteristic duration between 40 and 150 ms. This value is on the order of magnitude of fast sweeps over future spatial paths during rodent vicarious trial and error behaviour^31^ and of retrospective replay of human non-spatial paths^41,42^. Notably, these processes involve fixed sequences of more than two states, whereas in our case there were only two possible states such that any sequential outcome representation must be alternating. It remains to be shown whether representation of more than two possible action outcomes would follow a particular sequence, for example an ordering in terms of utility, probability, or previous encounters.

We have previously shown that in our task, cue-induced hippocampal gamma oscillations, and hippocampus-prefrontal cortex theta synchronisation, depend on loss probability^36^. In contrast, the current data suggest that outcome representation during choice deliberation depends on loss magnitude, but not on loss probability. Specifically, the positive outcome was more strongly represented when loss magnitude was lower. Since the occurrence of a positive outcome in our task depended on loss probability alone, whereas outcome representations were affected by loss magnitude alone, it appears that the neural outcome representations decoded here do not reflect a probabilistic model of the task structure. Instead, they might reflect a bias in memory recall that in turn instructs choice^3,19,20^. We note, however, that such model would also predict an influence of loss probability on outcome representation as well, something we did not find. As a limitation of our task, loss magnitude spanned over a larger range, had a larger influence on behavioural policy than loss probability (Table 2 and Fig. 1b), and was unambiguously signalled in the experiment, while loss probability had to be learned from experience and retrieved during action selection. Experiments manipulating loss magnitude and probability in more symmetric ways could help elucidating whether loss probability biases outcome representations in a similar manner as loss magnitude.

Positive outcome representation predicted approach from about 1400 ms after trial initiation (Supplemental Fig. 4). Immediately before an action (after token appearance), negative outcome representation predicted avoidance (Fig. 4). Intriguingly, this might relate to models in which value-based decisions are taken by sequential sampling from memory^30^, and to a model in which attentional mechanisms engender behaviour^3,19,20^. In particular, it is possible that differences in the representational strength of the two outcomes produce an effect comparable to an internal attentional bias, which in turn biases the evidence accumulation in favour of the options that is attended more^3,19,20^. Here, representations of the positive and negative outcome might serve as evidence in favour of approach and avoidance, respectively, suggesting that brain representations are in fact pieces of evidence recalled from memory. Finally, the observed effect of brain representations on behaviour is also in agreement with the representational bias towards desired goals observed in the forward sweeps of possible future paths by place cells activity in rodents^32,51^. The finding that outcome representations relate to behaviour suggests that their dynamics form an integral part of the decision process.

To decode outcome representations, we built classifiers on data acquired while participants were encountering the outcomes. Accuracy of the classification was enhanced by selecting training samples at the latency post-outcome where the group-level accuracy peaked (i.e., 310 ms; Fig. 2b). Instead of training each participant’s classifier at the participant-specific peaks, which would maximise classification accuracy, this approach allows for a straightforward interpretation of the classification results with respect to the source localisation (Fig. 2d) and previous literature.

Notably, this 310 ms peak is slightly later than the peak at 200 ms reported in previous MEG studies using similar methods^41,52,53^. A major difference between these previous studies and ours is that they employed highly differentiable visual stimuli, whereas stimuli in our task were visually relatively similar but had different valence. Instead, a latency of 310 ms is compatible with the P300 component of the event-related potential (ERP), which has been implicated in decision-making and stimulus evaluation^48,54^. In particular, the P300 is affected by the uncertainty associated with a decision^55^ and by the magnitude of the reward or loss coupled with a stimulus^56–58^. As a caveat, its amplitude also increases with the rarity of a stimulus^48,59,60^. Although in our task the negative outcome was rarer than the positive one, it is unlikely that our classification was predominantly based on surprise-related neural activity. In this case, more rare negative outcomes should improve classification compared to less rare negative outcomes, but we observed the opposite pattern (Fig. 2c). Interestingly, the baseline classification accuracy was slightly higher for higher loss probabilities. A possible explanation is related to the structure of our task. Catch probability increases with the time spent at the token position. Any neural signal that is related to motor performance would predict whether participants get caught or not, and may thus explain this above-chance accuracy already at baseline. Nevertheless, this slight increase in baseline classification accuracy is relatively small, compared with the accuracy that is reached when the actual outcome is displayed.

Previous studies have found that probability and reward/loss magnitude affect outcome-related ERP^56,57,61^, thus raising questions on whether our classification scheme generalises across experimental conditions. We addressed this with a cross-classification procedure: this was similar to the main analysis, with the difference that one level of probability/magnitude was left out of the training set and used as test set. We found accuracy to be comparable to the main analysis, in which classifiers trained with data from all conditions (Supplemental Fig. 2 and Fig. 2b). This suggests that our classifier captured features of the outcome representations are were largely invariant across loss probabilities and magnitudes in our task. The cross-classification analysis additionally supports the conclusion that high-loss probability increases outcome discriminability: excluding trials with high-loss probability reduced the classification accuracy more than excluding low probability (Supplemental Fig. 2, left), hence confirming that high-probability trials provide more information for discrimination.

While previous work on retrieval of action outcomes during choice deliberation has highlighted a role of the OFC^6,10–12,27^, there is also ample evidence of widespread representation in multiple brain areas during different phases of outcome anticipation^13^, including sensory cortices^43–45^. Therefore, we did not spatially constrain our analysis and let the classification capitalise on all the available sensors. Source reconstruction confirmed that the MEG patterns responsible for outcome classification were mainly generated in right prefrontal cortex including the OFC and dlPFC, as well as visual areas. In these prefrontal regions oscillatory power was higher for negative than positive outcome, in agreement with animal literature reporting stronger oscillatory activity in the prefrontal cortex during approach/avoidance conflict compared to familiar environments^62–66^. This rodent and related human work has additionally investigated the role of hippocampal oscillations^36,63,64^. However, decoding neural representations from source-reconstructed MEG data with hippocampal origin appears currently out of reach. Recent developments towards higher signal-to-noise ratios in human MEG, for example by restricting head motion^67–69^ or by using advanced sensor technology^70^, could help addressing the role of subcortical areas in such scenarios.

An open question is how representation of multiple possible outcomes is integrated to elicit choice, specifically regarding the population-level representation of the different outcomes^3,33^. Our MEG approach cannot differentiate whether sequential retrieval is instantiated in the same or different neural population. Possibly, functional magnetic resonance imaging repetition suppression could be leveraged to answer such questions^71,72^.

As a limitation, our approach of decoding representation of only two outcomes precludes a firm conclusion that decision-makers represent outcome identity, rather than one or several outcome features or dimensions. This concern is inherent in any decoding approach with a limited number of exemplars and independent of the data recording and analysis technique, such as MEG, local field potential, or single-unit activity.

To summarise, we provide evidence consistent with sequential neural representations of possible outcomes during probabilistic choice, with possibly stable representation epochs of duration between 40 and 150 ms. The prevalence of positive outcome representations depends on potential loss early during choice deliberation and predicts choice 1400 ms into the deliberation period. At the same time, negative representations depend on potential loss and predict choice immediately before choice execution. Our work furnishes a proof-of-principle that sequential representation of outcomes during probabilistic decision-making can be decoded from MEG signals during deliberation, and thus pave the way for more detailed investigation of the neural populations that carry out these operations.

## Methods

### Dataset

Twenty-three participants (22.9 ± 3.6 years; 14 female) were recruited from the general population. They were right-handed, fluent in English, reported no history of psychiatric or neurological disorder normal or corrected-to-normal vision. Two participants were excluded from the final analysis: one displayed large head motion (>0.5 cm) and the other one did not complete the experiment. All participants gave informed written consent before the beginning of the experiment. The study, including the form of taking consent, was conducted in accordance with the Declaration of Helsinki and approved by the University College London Research Ethics Committee. Source-space analysis of induced oscillations in this data set was published previously^36^.

### Experimental paradigm

The experimental paradigm was an approach/avoidance conflict test embedded in a computer game, in which participants pressed keys on a button box to control a virtual agent with the goal of collecting monetary tokens under the loss of virtual predation. A total of 576 trials were presented, divided into an initial training block of 36 trials, which was not analysed, and five subsequent blocks of 108 trials each: therefore, 540 trials were included in the final analysis. After the experiment, participants received financial compensation according to their performance in six randomly chosen trials (6 GBP for each collected token). Each trial started with the human player at the bottom block of a 2 × 2 grid arena and a virtual predator in the opposite grid block (Fig. 1a). As long as the player remained in this initial safe position, they were unreachable by the predator. After a random time interval (with duration equal to the minimum value from {6 s, *t*}, *t* being a random sample from a gamma distribution with shape parameter *k* = 2 and scale parameter *ϴ* = 1, resulting in a mean of 2 s), a token appeared in the left or right grid block. The token disappeared after another random time interval from the same distribution. While the token was in play, the player could collect it by moving from the safe position to the token position. This could lead to three possible outcomes: (1) a positive outcome P, if the agent returned to the safe position after collecting the token, (2) a negative outcome N, if the predator woke up and caught the agent, causing the loss of a variable number of tokens (between zero and five), or (3) a neutral outcome F if the player left the safe place but failed to collect the token because it disappeared before it was collected. These neutral outcomes were overall rare and not analysed (Table 2). Alternatively, the player could decide not to collect the token. The number of tokens that could potentially be lost was explicitly signalled in every trial and is referred to as loss magnitude. Three predators, signalled by the frame colour, differed in their wake-up probability (loss probability). This probability was not explicitly instructed but could be learned by the player during the initial 36 training rounds and throughout the task. Whether the predator would wake up was determined independently in every 20 ms time bin that the player spent outside the safe place as a Bernoulli event with probability of 0.02, 0.04, or 0.06, for the three different predators, respectively. For every 100 ms that the player spent outside the safe place, this resulted in a catch probability of ~0.1, 0.2, or 0.3, respectively. Colour/loss probability association was counterbalanced across participants. The trial ended 1000 ms after token disappearance, and was followed by a random inter-trial interval (ITI) drawn from the same gamma distribution regulating token appearance/disappearance, with a maximum of 4 s. In our analysis, we determined the neural representation of the outcomes P and N, and searched for these neural representations in two deliberation phases before action: directly at trial start, and shortly after token appearance.

### MEG data acquisition

MEG data were collected with a 275-channel Canadian Thin Film system with superconducting quantum interface device (SQUID)-based axial gradiometers. Data were hardware anti-aliased with cutoff frequency of 150 Hz and digitised at 600 Hz. Head positioning coils were attached to the nasion and left and right auricular sites, to provide anatomical coregistration and allow head localisation throughout the experiment. Trial onset, token appearance, and trial end times were written into the MEG data via a TTL parallel port. The computer game was projected on a screen positioned ~0.8 m from participants’ head. Participants controlled the virtual agent with a button box.

### MEG data preprocessing

**Fig. 5:**
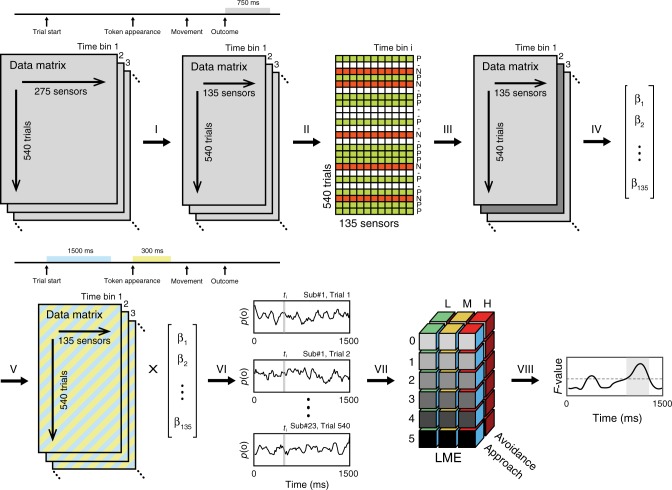
Multivariate data analysis. Classifiers were trained on the MEG field patterns acquired while participants were experiencing the outcomes (grey segment). For each participant, this was a data structure containing the magnetic field at each of the 275 sensors acquired in each of the 540 trials. (i) Channel selection. The number of channels was reduced to 135 by selecting the participant-specific subset containing the least eyeblink artefacts^41^. (ii) We then computed the cross-validated accuracy of the classification at each time bin. As training set, we retained approach trials in which either the positive (P, green) or the negative (N, red) outcome was presented, and discarded neutral and avoidance trials (hyphen, white). At this stage the regularisation *λ* coefficient was set to 0.025. (iii) The time bin of peak accuracy was then selected to build the training set of the classifiers, which (iv) were defined as the 135 weights associated with each channel resulting from a lasso-regularised logistic regression. The *λ* coefficient used at this stage was optimised with a second cross-validation procedure. (v) Analysis of the deliberation phase: the classifiers were then used to estimate the relative probability that either outcome was being represented during deliberation aligned either to trial start (cyan segment) or token appearance (yellow segment). (vi) The classification resulted in outcome representation probability (*p*(o)) time series of which we considered one time bin at the time to (vii) compute a LME and extract fixed-effects statistics (loss probability: low (L), medium (M), high (H); loss magnitude: 0–5; choice: approach, avoidance). (viii) For statistical inference, we applied a non-parametric cluster-level correction over the *F*-values of the main effects resulting from the LME.

MEG preprocessing was done in SPM12 (Statistical Parametric Mapping, Wellcome Trust Centre for Neuroimaging, London, UK, www.fil.ion.ucl.ac.uk/spm). Continuous raw MEG data were high-pass filtered with a cutoff frequency of 0.5 Hz to remove slow signal drifts, notch-filtered at 50 Hz to remove mains noise, and down-sampled to 100 Hz. In order to reduce the potential effect of eyeblink artefacts, we followed a conservative approach used in previous MEG work^41^ and retained the participant-specific set of 135 channels containing the fewest eyeblink artefacts across the entire time series (Fig. 5(i)), as determined by the SPM12 eyeblink artefact detection algorithm. For most participants, this resulted in the exclusion of occipital and frontal channels (Fig. 2a). To validate this method on our data, we compared it with a topography-based artefact correction algorithm implemented using SPM. After computing the average shape of the artefact, the method reconstructs the topography of the artefact, and later corrects the data features that match such topography. Since the two returned very similar results, we here report the simpler approach of retaining the 135 cleanest channels.

We then extracted epochs (with no baseline correction) from 0 to 1500 ms after trial start (first deliberation epoch), from 0 to 300 ms after token appearance (second deliberation epoch), and from 0 to 500 ms after the onset of the decision outcome. The onset of the positive outcome P coincided with the time at which the agent secured a collected token by moving back to the safe place, whereas the onset of the negative outcome N was identified as the time at which the predator caught the agent. We discarded all trial start epochs during which the token appeared within 1500 ms from trial start (as well as a small number of trials during which the agent moved before token appearance within this time window), and all token appearance epochs during which an action occurred before 300 ms from token appearance. Hence, all analyses of the three epoch types were performed on non-overlapping data sets.

### Multivariate data analysis

We sought to determine how action outcomes were represented during the deliberation phase of each trial. Our analysis pipeline is illustrated in Fig. 5, and was inspired by a previous publication on visual outcome representations in a non-spatial reasoning task^41^. We focused on the two possible outcomes of an approach action. The outcome of the other action, avoidance, was a continuation of the current state and not locked to a specific moment in time.

### Determining neural representation of action outcomes

We first determined the neural representations of the two action outcomes: successful collection of the token (P) or catch by the predator (N). To this end, we created binomial pattern classifiers of the MEG activity during the 0–750 ms after outcome presentation. For each participant and trial, these data consisted of a time series of 75 time bins for each of the 135 retained MEG channels. The participant-specific number of trials used to train the classifier depended on the number of approach responses, and this was (mean ± standard deviation) 374 ± 81. The mean ratio between the two action outcomes P and N probabilistically depended on the participant’s return times and was 5.6 ± 1.8. Data from each time bin were extracted and labelled according to whether an approach response was followed by P or N, while trials with neutral outcome and avoidance responses were discarded (Fig. 5(ii)). Classifiers were built by applying the lasso-regularised logistic regression function lassoglm implemented in MATLAB on these labelled data. To compute the relative accuracy, the *λ* coefficient of the lasso regularisation, which determines the penalty for each non-zero coefficient, was initially set arbitrarily to 0.025. We then used a cross-validation procedure to determine the time bin after the onset of the outcomes that maximised classifiers’ aggregate performance (Fig. 5(iii)) estimated in terms of the balanced accuracy, defined as

1 $$\frac{1}{2}\left( {\frac{{{\mathrm{True}}\,{\mathrm{positives}}}}{{{\mathrm{All}}\,{\mathrm{positives}}}} + \frac{{{\mathrm{True}}\,{\mathrm{negatives}}}}{{{\mathrm{All}}\,{\mathrm{negatives}}}}} \right)$$

Next, labelled data from the optimal time bin were used to re-compute the classifier (Fig. 5(iv)); this time, the *λ* coefficient was left free and optimised.

To perform the cross-classification (Supplemental Fig. 2), we first separated the trials according to loss probability or magnitude. For each participant, we then created classifiers in which one level of either manipulation was left out and used as the test set, while all the other trials were used for training. Therefore, we had a total of nine classifiers per participant (one for each of the three loss probabilities and six loss magnitudes). We then computed the group-level accuracy of these nine classifiers and tested their statistical significance with a one-sided Wilcoxon signed rank test—a non-parametric statistical that relaxes the normality assumption that is violated by bounded variables like classification accuracies.

Since there were only two possible outcomes, we initially used a binomial classifier to compute the probability of P and N. Hence, during analysis of the deliberation phase, this artificially imposed a constraint that only one of these two outcomes is represented. If an experimental manipulation led to a stronger representation of one outcome over the other, it remained unclear whether this arose from a stronger representation of one outcome or weaker representation of the other. To disambiguate this, we created two additional binomial classifiers to distinguish either outcome from a baseline. One hundred baseline examples were taken at random time bins during the 1000 ms preceding trial start. To avoid interferences from the previous trial, baseline examples were extracted only from ITI longer than 2000 ms. To summarise, we trained a total of three classifiers per participant: (a) P vs. N (b) P vs. baseline and (c) N vs. baseline. For each of these classifiers, we created a set of 100 additional classifiers after random permutation of the outcome labels to create null distributions for statistical testing. These are referred to as permuted classifiers.

### Searching for neural representation of action outcomes during deliberation

We applied these classifiers to MEG activity at each time bin during the two deliberation epochs (Fig. 5(v)). From the pattern of 135 channel signals at each time bin (cyan and yellow segments, Fig. 5), a probability was obtained by multiplying element-wise these signals with the corresponding weight of the classifier and then mapping the result onto the interval [0,1] with the standard logistic sigmoid function $f\left( x \right) = \frac{1}{{1 + e^{ - x}}}$ (Fig. 5(vi)).

### Autocorrelation

To study the temporal structure of the outcome representations, we computed the autocorrelation of the decoded probabilities. To assess whether they differed from chance, we compared the autocorrelation against a null distribution created at each time lag from the 100 permuted classifiers. Specifically, the likelihood of the autocorrelation under the null distribution at a given time point was approximated to the relative number of permutations that resulted in a more extreme (two-sided) value for the autocorrelation. Clusters were defined as the sets of consecutive time points for which the log-likelihood (LL) was larger than 3, and cluster size was quantified as the sum of the LL of all the points in the cluster. We performed group-level statistics at the cluster-level with a non-parametric permutation test, and report only the clusters that were bigger than the biggest cluster found in 95% of analyses with the permuted classifiers^50^. Note that this test controls the false positive rate across the entire time interval; the location of clusters is reported for illustration only. Next, we collapsed the decoded probabilities into the most likely represented outcome (i.e., P if *p*(P) > *p*_chance_; N if *p*(P) < *p*_chance_, where *p*_chance_ was determined at the participant level by the relative number of occurrences of positive and negative outcomes in the training set). We then took the resulting set of epochs of steady representation (i.e., the time interval during which the most likely represented outcome did not change) and computed the distribution of their duration. This distribution was tested against the same distribution computed from the permuted classifiers. Statistical difference was tested with a two-sample Kolmogorov–Smirnov test. We also tested the average number of transitions from one outcome representation to the other (i.e., number of epochs) against the number of transitions predicted under the null distribution obtained from the permuted classifiers. The *p*-value was computed as the proportion of more extreme results from the permuted classifiers.

### Source reconstruction

To explore the neural underpinnings of outcome evaluation, we used a beamformer spatial filtering algorithm, which estimates the distribution of underlying sources. To generate the MEG forward model, we used the Montreal Neurological Institute (MNI) template brain, and a single-shell head model. The MNI template was coregistered using the nasion, left and right preauricular points as fiducial points. We then applied the beamforming algorithm at a temporal window of 260–360 ms after outcome presentation and a frequency range of 1–50 Hz. We chose these parameters in order to match as closely as possible the features that our classifier was trained on (i.e., a 100-ms window centred around the latency of peak accuracy at 310 ms, and using the full frequency spectrum that is preserved in the 100 Hz sampled MEG signal). For each participant, the beamforming algorithm generated three-dimensional source power images for P and N on a 5 mm grid and smoothed with a Gaussian Kernel with full width at half maximum (FWHM) of 10 mm. Single-participant contrasts were then computed as difference maps P – N and N – P, and were finally tested for statistical significance at the group level with one-sample *t*-tests and whole-brain corrected for family-wise error at *p* < 0.05.

### Statistical analysis

Next, we sought to estimate the effect of loss probability and loss magnitude on outcome representations *p*(*R*_O_), with O = P, N, and whether they were predictive of behaviour. To this end, we sought to test how the probability of outcome representations during deliberation varies with loss probability, loss magnitude, or ensuing choice. To do this, we fitted the inverse sigmoid of the probability *p*(*R*_O_) at each time point with a linear mixed models (R function lmer, lme4 package) on the aggregate data, as in our previous works^34,36^. The advantage of these models is that they provide meaningful parameter estimation even with unbalanced data sets^73^, such as the one used in this study, where assumptions of repeated-measures analysis of variance (ANOVA) are violated. We first applied an inverse sigmoid to our data, so that

2 $$Y = \ln \left( {\frac{{p(R_{\mathrm{O}})}}{{1 - p(R_{\mathrm{O}})}}} \right),\quad {\mathrm{O}} = {\mathrm{P}},{\mathrm{N}}.$$

The model had the following form

3 $${Y = \beta _0 + {\mathop {\sum}\limits_{i = 1}^3}\, {\beta _iX_i} + {\mathop {\sum}\limits_{i = 1}^2}\, {\mathop {\sum}\limits_{j > i} {\beta _{ij}X_iX_j} } + \beta _{123}X_1X_2X_3 + b_k + {{\it{\epsilon }}}}\\ {b_k \sim N\left( {0,\sigma _b^2} \right),\,k = 1 \ldots n} \\ {{\it{\epsilon }} \sim N\left( {0,\sigma ^2} \right).}$$

In the above formula, *β*_0_ is the group intercept, *b*_*k*_ the random subject intercept, *β*_*i*_ is the fixed main effect of factor *i* (loss probability, loss magnitude, or behaviour), and *β*_*ij*_ and *β*_123_ are the two- and three-way interactions, respectively. This is equivalent to the R formula

4 $$Y \sim {\mathrm{loss}}\,{\mathrm{probability}}\, *\,{\mathrm{loss}}\,{\mathrm{magnitude}}\, * \,{\mathrm{behaviour}} + \left( {1{\mathrm{|}}{\mathrm{subject}}} \right).$$

Fixed effect *F*-statistics on the fitted parameters were computed with the R function anova (Fig. 5(vii)). The number of degrees of freedom used to compute the *p*-values was conservatively set to the lower bound of the effective degrees of freedom of the denominator

5 $${\mathrm{df}} = N - K,$$

Where *N* is the number of observation and *K* is the number of all the fixed and random effects in the model. Multiple comparison correction was performed with a non-parametric permutation test on the cluster level (inclusion threshold *p* < 0.05; Fig. 5(viii))^50^.

For the behavioural analysis (which was already reported in our previous study^36^), we used a similar linear mixed effects model as above, using the model formula

6 $$Y \sim {\mathrm{loss}}\,{\mathrm{probability}} \, * \,{\mathrm{loss}}\,{\mathrm{magnitude}} + \left( {1{\mathrm{|}}{\mathrm{subject}}} \right),$$

together with an identity link function for approach latency and a logistic link function for approach action.

### Reporting summary

Further information on research design is available in the Nature Research Reporting Summary linked to this article.

## Supplementary information

Supplementary Information

Reporting Summary

## Data Availability

Data are available from the authors upon reasonable request due to ethics restrictions.

## Code availability

For data collection, we developed a computer task with MATLAB and the MATLAB toolbox Cogent 2000. To analyse the data, we used MATLAB with the toolbox SPM12 and its extension DAiSS, and custom code. For linear mixed-effect models for inferring the effect of experimental manipulations on representation probabilities, we used R and its toolbox LME4 (version 1.1–13). The codes used for data collection and analysis are freely available for download from the GitHub page of our research group (https://github.com/bachlab/megaa).
