# Supplementary material for: Representation of probabilistic outcomes during risky decision-making
Source: Nat Commun. 2020 May 15;11:2419. doi: 10.1038/s41467-020-16202-y (PMC7229012; doi:10.1038/s41467-020-16202-y)
Supplement: Supplementary file 1 — Supplementary Information [file 41467_2020_16202_MOESM1_ESM.pdf]

Supplemental Figures

Representations of probabilistic outcomes during risky decision making

Castegnetti et al., 2020

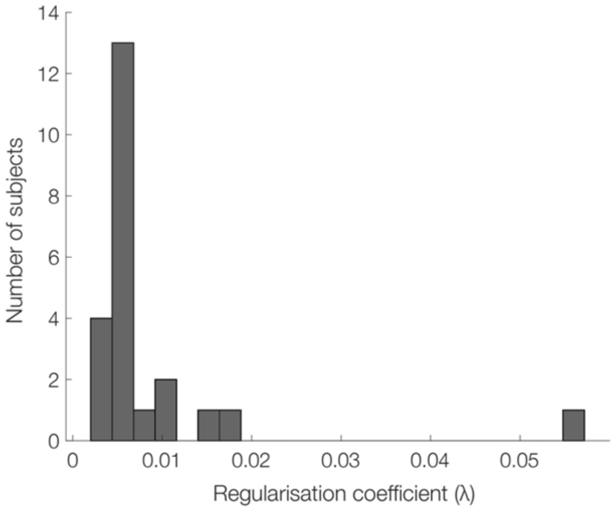

Supplemental Figure 1. *Group-level distribution of λ coefficients used to lasso-regularise the P vs. N classifiers.*

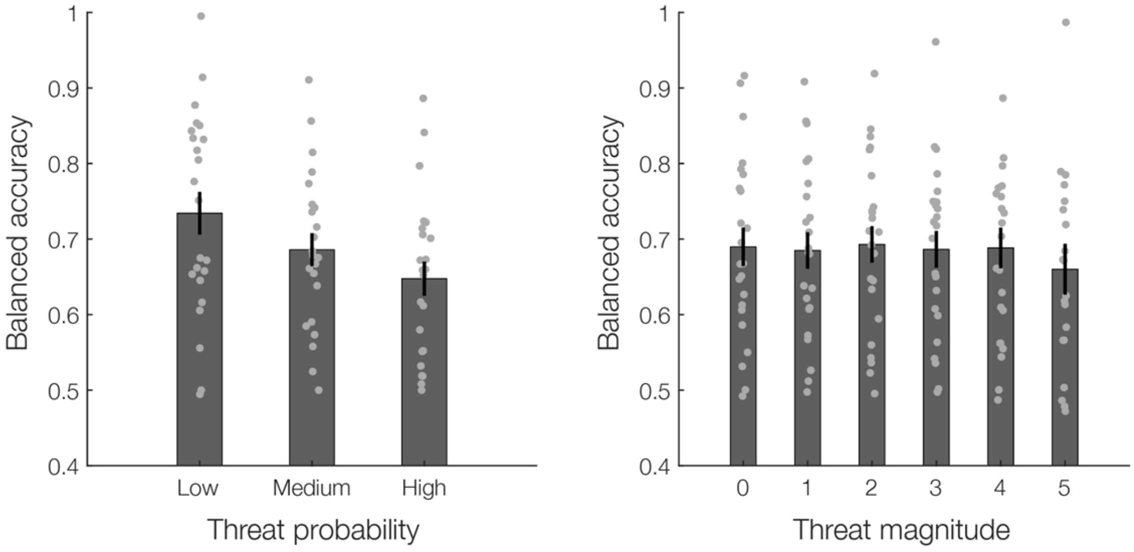

Supplemental Figure 2. *Balanced accuracy (chance level: 0.50) obtained from the cross-classification procedure. For each loss probability and magnitude, the dots indicate the individual accuracy for each participant (n=21), the bar indicates the average accuracy of a classifier, trained on all other probabilities and magnitudes and tested on the probability/magnitude itself, the vertical bar indicates the standard error of the mean (s.e.m.). All accuracy were above chance with p < 0.001 (one-sided Wilcoxon signed rank test).*

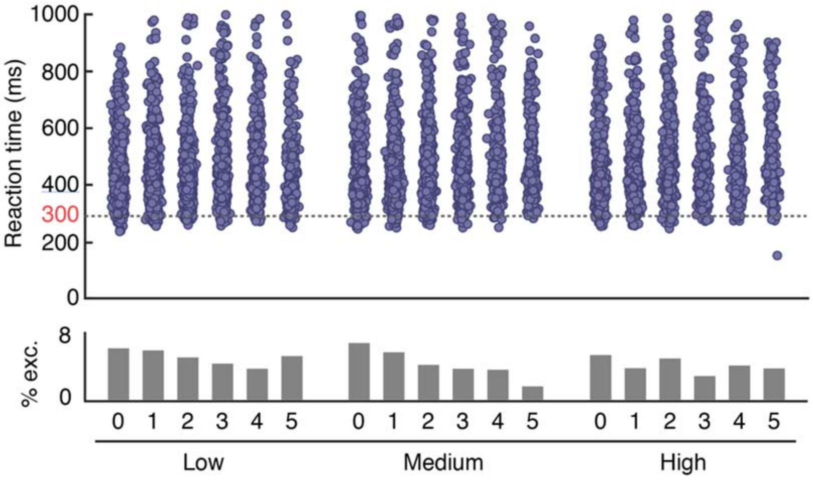

Supplemental Figure 3. *Top: Reaction times for each loss probability and magnitude for each trial from all participants. Each dot represents an individual trial. Trials with reaction time shorter than 300 ms (horizontal dashed line) were excluded from the analysis, resulting in the exclusion percentage indicated in the Bottom panel (< 8% in all cases).*

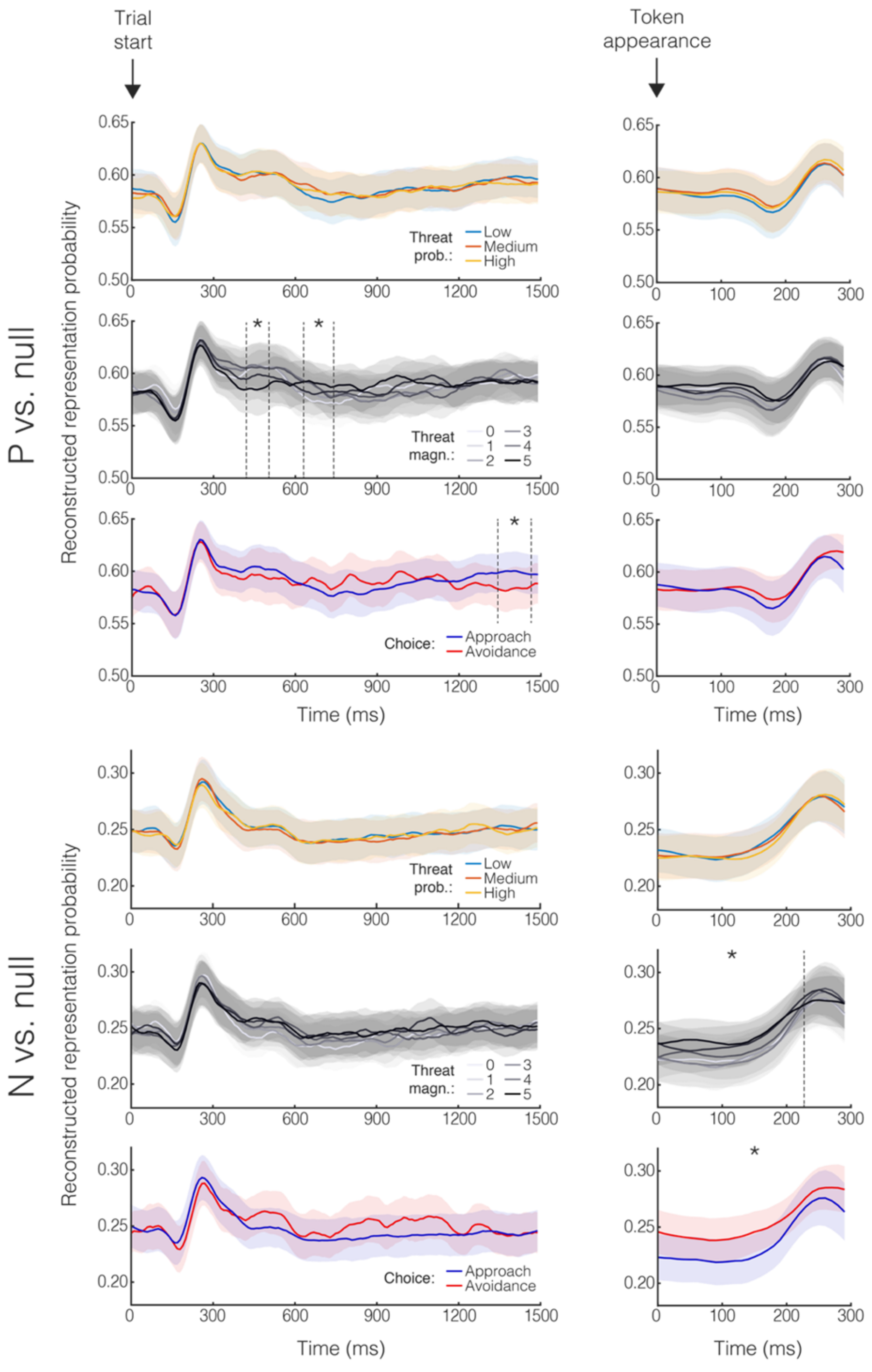

Supplemental Figure 4. *Reconstructed outcome representation probabilities, analogous to Figure 4 in the main text (where we classified P from N) but for the classifying P or N from baseline. Displayed are the probabilities that the MEG field patterns during deliberation represents P (top six panels) or N (bottom six panels) and their s.e.m. (coloured areas), for epochs between 0 and 1500 ms from trial start and between 0 and 300 ms from token appearance (note the different time scales in plots aligned to trial start or token appearance). Intervals denoted with * illustrate epochs in which the effect of the corresponding manipulation was statistically significant in a cluster-level random permutation test across the entire epoch.*
