# Supplementary material for: Representation of probabilistic outcomes during risky decision-making
Source: Nat Commun. 2020 May 15;11:2419. doi: 10.1038/s41467-020-16202-y (PMC7229012; doi:10.1038/s41467-020-16202-y)
Supplement: Supplementary file 2 — Reporting Summary [file 41467_2020_16202_MOESM2_ESM.pdf]

nature research

Corresponding author(s): Giuseppe Castegnetti

Last updated by author(s): Mar 26, 2020

Reporting Summary

Nature Research wishes to improve the reproducibility of the work that we publish. This form provides structure for consistency and transparency in reporting. For further information on Nature Research policies, see Authors & Referees and the Editorial Policy Checklist .

Statistics

For all statistical analyses, confirm that the following items are present in the figure legend, table legend, main text, or Methods section.

n/a	Confirmed	
☐	☒	The exact sample size (*n*) for each experimental group/condition, given as a discrete number and unit of measurement
☐	☒	A statement on whether measurements were taken from distinct samples or whether the same sample was measured repeatedly
☐	☒	The statistical test(s) used AND whether they are one- or two-sided<br>*Only common tests should be described solely by name; describe more complex techniques in the Methods section.*
☐	☒	A description of all covariates tested
☐	☒	A description of any assumptions or corrections, such as tests of normality and adjustment for multiple comparisons
☐	☒	A full description of the statistical parameters including central tendency (e.g. means) or other basic estimates (e.g. regression coefficient) AND variation (e.g. standard deviation) or associated estimates of uncertainty (e.g. confidence intervals)
☐	☒	For null hypothesis testing, the test statistic (e.g. F, t, r) with confidence intervals, effect sizes, degrees of freedom and P value noted<br>*Give P values as exact values whenever suitable.*
☒	☐	For Bayesian analysis, information on the choice of priors and Markov chain Monte Carlo settings
☐	☒	For hierarchical and complex designs, identification of the appropriate level for tests and full reporting of outcomes
☒	☐	Estimates of effect sizes (e.g. Cohen's *d*, Pearson's *r*), indicating how they were calculated

Our web collection on statistics for biologists contains articles on many of the points above.

Software and code

Policy information about availability of computer code

Data collection	For data collection, we developed a computer task with MATLAB and the MATLAB toolbox Cogent 2000. The task scripts are publicly available from the research group's GitHub page (https://github.com/bachlab/megaa).
Data analysis	To analyse the data, we used MATLAB with the toolbox SPM12 and its extension DAiSS, and custom code. Linear mixed-effect models for inferring the effect of experimental manipulations on representation probabilities used R and its toolbox LME4 (version 1.1-13). Data analysis scripts are publicly available from the research group's GitHub page (https://github.com/bachlab/megaa).

For manuscripts utilizing custom algorithms or software that are central to the research but not yet described in published literature, software must be made available to editors/reviewers. We strongly encourage code deposition in a community repository (e.g. GitHub). See the Nature Research guidelines for submitting code & software for further information.

Data

Policy information about availability of data

All manuscripts must include a data availability statement. This statement should provide the following information, where applicable:

- Accession codes, unique identifiers, or web links for publicly available datasets
- A list of figures that have associated raw data
- A description of any restrictions on data availability

The data are available from the authors upon reasonable request due to ethics restrictions.

Field-specific reporting

Please select the one below that is the best fit for your research. If you are not sure, read the appropriate sections before making your selection.

Behavioural & social sciences study design

All studies must disclose on these points even when the disclosure is negative.

Study description	The study was aimed at investigating the neural responses to risky decision making from quantitative behavioural and quantitative magnetoencephalograpy (MEG) data.
Research sample	Twenty-five participants (22.9 ± 3.6 years; 14 female) were recruited from the general population in London, UK.
Sampling strategy	Participants were sampled by public advertisements. Since this manuscripts presents a re-analysis of an existing data set, sample size was not based on a formal power analysis.
Data collection	MEG data were collected with a 275-channel Canadian Thin Film system with superconducting quantum interface device (SQUID)-based axial gradiometers. The experiment was presented on a screen, with no direct interaction between experimenter and participant.
Timing	03-04/2014
Data exclusions	Two participants were excluded from the final analysis: one displayed large head motion (> 0.5 cm) and the other one did not complete the experiment. The exclusion criteria for head motion were established at the beginning of the study.
Non-participation	One participant did not complete the experiment because they felt unwell in the MEG scanner.
Randomization	Participants were not allocated into experimental groups.

Reporting for specific materials, systems and methods

We require information from authors about some types of materials, experimental systems and methods used in many studies. Here, indicate whether each material, system or method listed is relevant to your study. If you are not sure if a list item applies to your research, read the appropriate section before selecting a response.

Materials & experimental systems

n/a	Involved in the study	
☒	☐	Antibodies
☒	☐	Eukaryotic cell lines
☒	☐	Palaeontology
☒	☐	Animals and other organisms
☐	☒	Human research participants
☒	☐	Clinical data

Methods

n/a	Involved in the study	
☒	☐	ChIP-seq
☒	☐	Flow cytometry
☒	☐	MRI-based neuroimaging

Human research participants

Policy information about studies involving human research participants

Population characteristics	We recruited twenty-five participants (22.9 ± 3.6 years; 14 female) from the general population in London, UK. They were right-handed, fluent in English, reported no history of psychiatric or neurological disorder and had normal or corrected-to-normal vision.
Recruitment	The participants were recruited from online platforms that target the student and general population. Due to the procedures used to advertise the study, we mainly recruited students and young adults.
Ethics oversight	The study was conducted in accordance with the Declaration of Helsinki and approved by the University College London Research Ethics Committee.

Note that full information on the approval of the study protocol must also be provided in the manuscript.
